# Some of the Physical Properties of Metals

**Published:** 1896-06

**Authors:** A. A. Breneman


### SOME OF THE PHYSICAL PROPERTIES OF METALS.[1]

BY PROFESSOR A. A. BRENEMAN.[2]

It has been well said that science is only "educated common sense," and there is nothing in science that is valuable in practice which cannot be told in language quite plain to intelligent persons outside of the given field of science. The subject for this evening, "Some Physical Properties of the Metals," is one of very wide range, so I shall limit my talk to a consideration of some of the physical properties of the more familiar metals, especially such as you in your work are accustomed to deal with. The term metal itself is difficult to define. A well-known authority on this subject has said that there is no representative metal,—that is, that no one metal combines in itself all the qualities which occur to us when we use the term metal; the only typical metal is an ideal metal.

Before going into a consideration of the properties of metals on which I expect especially to dwell, I will first ask your attention to a matter of theory. Theory in chemistry and physics is something like diagnosis to the physician, it outlines the subject and furnishes a bases of treatment. Unlike diagnosis in its limited meaning, a given theory may furnish a working hypothesis for the entire science. The method of scientific progress within the age in which science as such can be said to have had a real existence, has been that of fixing upon some interpretation of facts already at hand, and upon the basis of this interpretation of facts constituting a theory by which to devise experiments that will act as a test of the theory, and serve also as a means of discovering new facts. Every theory starts upon a very small basis, a mere guess may be the beginning of it, but science is not content with guesses. The guess must be tested by experiment. The guess serves only to suggest an experiment, and the result of the experiment is itself a test of the value of the guess. Add together many guesses or speculations, and many experiments testing the validity of these speculations, and we have the basis of a theory. It has been said that in science one "guesses and checks his guesses." In this matter of devising experiments which shall serve as a test of speculation, imagination itself is an important factor. The mind must move through all the materials it has at hand and pick from them

---

[1] Read before The New York Institute of Stomatology, April 7, 1896.

[2] Late Professor of Industrial Chemistry in Cornell University.

a suggestion for its second step. Many years ago Professor Tyndall used the expression, "the scientific use of the imagination," and wrote under that title an essay which will always be regarded as a classic; yet in his day Professor Tyndall was taken to task by shallow people outside of science for the use of the term, and it was claimed to be an admission that, after all, the matter of science was largely imagination. No greater mistake could have been made. The meaning of Tyndall was that the imagination must be used for its suggestiveness in devising experiments. After that, experiment will settle the value of the suggestion. You see, then, that the imagination in this sense, while it is given an important place, is entirely subordinate to the principal work of science.

Now, in considering any form of matter we must know something about all conditions in which matter can occur. Matter, as you know, can take any of the three states,—solid, liquid, or gaseous; and we shall find that our familiar substances, the metals, can occur in all of these conditions. In fact, any metal can be made to take the state of a solid, a liquid, or a gas by changes of temperature merely. There is a well-known liquid metal, mercury, and there is good reason for believing that the lightest of all gases, hydrogen, is itself a metallic body. That most metals, however, are solids is familiar enough to you. It has always been my experience that in speaking of the properties of matter, and in thinking of them, one works with more system and with more effect if he has at hand an intelligible theory of matter. What is the difference in these three different states of matter; and why should a solid, a liquid, and a gas have each their distinctive properties? Chemists and physicists have long ago come to the conclusion that, in the main, a theory of matter which regards it as made up of distinct and very minute particles serves best to explain the properties of matter. There are exceptions to this, of course; there are other theories which have had a certain support in all ages, but the working theories of physical science deal mainly with the atom and the molecule as the units of matter. By an atom we mean an exceedingly small particle of matter. Primarily the term means something which cannot be further divided, but the discussion of the definition in this sense is merely a metaphysical one and need not concern us here. Let us assume that somewhere, in subdividing matter, we come to a practical unit,—the smallest particle. It is further believed that these atoms of which simple elements are composed can unite and form groups of greater or less size, and these groups, whether made of the same kind or different kinds of matter, we call molecules. There is good reason for believing, although it cannot as yet be proved, that there is, after all, but one form of matter, and that the seventy odd elements which the chemist knows of as distinct forms of matter may themselves be merely groupings, of more or less complexity, of one primary matter.

To deal now with these units of matter, the atom and the molecule, let us consider first the state of gas. A gas is believed to consist of particles of matter separated by distances much greater than their own diameters, which particles have a free motion that carries them forward in straight lines until they are arrested by contact with other atoms or with other forms of matter. In a liquid body the particles differ principally in that they have less freedom of motion. A particle of gas, we believe, might fly indefinitely in a straight line if not arrested. A particle of liquid seems to have somewhat the motion of a pendulum; if not a direct to-and-fro motion, at least a motion in which the particle returns, after a path of greater or less complexity, to the point at which it started. A liquid, therefore, while it has no definite shape, has yet a power of holding together, as may be seen when it forms drops. Mercury when spilled upon a plane surface breaks into little globules, and below a certain size the particles of mercury show a strong tendency to hold together. This tendency to hold together is only an expression of the fact that all the particles of this mass are confined to a certain sphere of motion and cannot move away from one another indefinitely, as the particles of a gas do. In the solid state it is believed that the difference is merely one of less freedom of motion permitted to the particles. The molecules of a solid have probably the same pendulum-like motion, but in much shorter paths than those of a liquid, and the distances between different molecules are evidently much less. If you will keep in your minds these rough distinctions between the three conditions of matter as they affect the molecules or smallest particles of a mass, you will have a mental picture which will enable you not only to recall the properties of matter more readily, but to explain the behavior of matter under given conditions when you have occasion to think of it.

Let us consider the properties of the metals themselves. One of the most evident properties of the metals, one in which they differ widely in degree, is that of hardness. Consider the range of hardness exhibited between the liquid metal mercury, or the metals sodium and potassium, which can be worked like putty between the fingers, and the hard metal iridium, or its alloys, which are used for giving points to gold pens. Or consider, again, the hardness of steel, a form of iron differing only from iron in having a certain proportion of carbon in combination. Why should one metal be hard and another soft? It is not a question merely of weight, because the softest of all metals, mercury, is one of the heaviest. I think you will see that the hardness of a metal depends in some way upon the difficulty of pushing the molecules apart. The point or edge with which you try to scratch the metal cannot enter it because the molecules of the metal persist each in following out its little path and refuse to be pushed out by the foreign substance used to test its hardness. It is the motion of the atoms which resists the point that tries to penetrate the mass.

Closely related to the property of hardness is that of tenacity. By tenacity we mean the power of resisting rupture. The tensile strength of a metal is measured by shaping it into a wire or rod of known cross-section, and then noting the force exerted at the moment when that rod or wire is broken apart by pulling upon it. Tenacity depends again upon the power of the molecules in persisting in their original paths. Anything which separates one portion of the mass of metal from another must, to some extent, interfere with the motion of the molecules as it existed previous to rupture.

Malleability is a property which is closely related to tenacity. We mean by the term malleability simply the property of flattening out under the hammer. Gold is an eminently malleable metal, it can be beaten into thin leaves. A metal which is spread out into a thin sheet under the hammer evidently suffers a violent displacement of many of its molecules; many of them are forced to take up new positions entirely different from those which they first occupied; and while their motion may not be greatly affected in the new position, a great deal of friction has occurred during the change, and this friction is made evident by the amount of heat set free during the change of position of the particles.

Brittleness is the reverse of malleability. While the majority of metals tend rather to malleability than to brittleness, yet there are marked cases of this second property. Antimony and bismuth fly to pieces under the hammer. Even zinc, at certain temperatures, is as brittle as glass and can be ground into a powder. Ductility is a property closely related to malleability. It is the quality which permits a metal to be drawn out into wire. This property depends again upon the persistence of the molecules in their chosen paths, their power of holding to those paths and to one another.

Whether this is a quality of actual attraction of particle to particle, as iron is attracted by a magnet, or whether it may not be some interlacing of the paths of oscillation of each particle, which links the mass together in a certain way, it is impossible to say.

Of the property just mentioned there are applications in the arts which rest upon such explanations as have been given. The drawing of wire, the spinning of metals upon a lathe, the spinning of a sheet of metal into the form of a hollow vessel through its tendency to escape from the point at which it is pressed between a hard surface and a hard tool, and the so-called squirting of metals under pressure, are all related to the foregoing properties. The squirting of a metal is effected by confining a soft and malleable metal in a cylinder, where it receives the pressure of a piston, while at the same time it has no means of escape from this pressure except through a small orifice in the bottom of the cylinder. Under these conditions the metal escapes through the small opening in the shape of a wire, or ribbon, or tube, according to the shape of the opening, and behaves as it passes through the opening as if it were in a liquid state. It is evident that the particles escaping through the orifice have moved very widely from their original position in the mass. It is evident, also, that as the piston descends in time so as to expel all or nearly all of the metal, that all of the particles originally in the mass must have suffered great changes in position. We speak in such cases of the metal flowing; and, doubtless, as in the flow of a stream of water, the motion is greater about the centre of the mass, and less along the sides. But at any given moment the motion at the orifice is evidently much greater than at any other point of the mass. Here there has been imparted to each particle, besides its own proper motion, which may have persisted all the time, a very large motion of displacement; it has been carried along as it moved somewhat as the moon moves in its own path around the earth and yet moves with the earth through space. The amount of pressure required, the degree of friction, and, therefore, the quantity of heat set free,—all of these depend originally upon the nature of the metal itself. The special form of motion which its particles originally had, the distance of those particles from one another, and probably also the weight of those particles, are all elements of their behavior. Yet by this analysis you can form a certain picture of the moving particles as the piston descends and the mass in the form of wire escapes from the opening.

The mobility of metals is a property nearly connected with those just treated of. I have said that a gas is perfectly mobile. In liquids we have very different degrees of mobility. The light liquid, ether, moves readily in a containing vessel, much more so than water. Mercury, although a much heavier liquid, is extremely mobile. A basin of mercury from which light is reflected so that the reflected light can be measured by the motion of a bright spot over a graduated scale is the most delicate instrument for recording motions too small for the unaided eye to see, and a surface of mercury is a most ready means of registering very slight earthquake tremors.

The flow of metals under such stress as is given in hammering or wire-drawing, or squirting, depends largely upon their mobility. Furthermore, when metals are thus changed in shape under the action of forces they seem to undergo a change which leaves them permanently altered under ordinary conditions; although this change can be annulled by very simple means. It is as if the particles at first violently crowded out of their places had not been left room for that free motion which they originally had, but are under a condition of constraint. Suppose that at certain points of the mass they are crowded together so that the original length of their path is not permitted. This would evidently produce some difference in quality, because the structure of the mass is not what it was at first. In practice we know that the mere device of heating a metal so strained or warped, for a considerable time, to a temperature below its melting-point will bring it back to its original condition, so that it will show again the same malleability, ductility, etc., as before; whereas, under the temporary strain it may have been harder or more brittle than at first. In the beating out of metallic foils, or in the drawing of wire, it is necessary to stop from time to time and anneal the metal,—that is, heat it to a certain temperature and allow it slowly to cool. Under these conditions there seems to be given that opportunity for the particles to return from their cramped positions to the original freedom of motion, and the metal regains its former properties. In the harder metals, such as iron and steel, the term *fatigue* is used to express the condition of strain under which the metal exists after the action of force upon it; and the fact that under the normal conditions it will in time regain its original properties quite justifies the use of the term fatigue.

In their behavior under heat metals exhibit the greatest variety of action. Mercury is a metal liquid at common temperatures; it is melted at those temperatures. We have only to cool it to 40° below zero and it becomes a solid body. And so we have among the class of metals every variety of fusibility, from mercury at the lower limit to such metals as chromium and platinum at the higher. One melts below the heat of the hand, others only at a temperature of the oxyhydrogen blow-pipe. Inasmuch as all matter expands under the action of heat,—that is, extends its boundaries in all directions,—we are led to believe, upon our present theory of matter, that the effect of heat upon its particles is simply to make them move with more force, and therefore through wider limits. It would be expected, therefore, that when a solid body such as a metal is sufficiently heated, its particles would push themselves apart until the distance between them was so great, and their own freedom of motion so great, that the solid state would be lost and a liquid would result. And if the temperature were carried still further, a further increase of motion would result finally in absolving all the particles from their attraction for one another, and from their motion, perhaps, in fixed paths, and would give us in time the perfect freedom of motion which exists in a gas. Solid, liquid, and gas, therefore, are simply three states of matter differing in the heat or pressure which has been applied to them; and there is no substance which cannot be brought, at a sufficiently low temperature, into the state of a liquid or a solid, or at the other extreme of temperature, into the state of a gas. And we may go further and say that there appears to be certain analogy between this uniform change of state which occurs under the action of heat and the peculiar and partial changes which occur when malleable or ductile metals are submitted to pressure or tension. Possibly the effect of force at the point where it is applied is such as to throw the particles immediately about that point into violent motion, or to give them that great freedom of motion which corresponds to the liquid condition. It has been very shrewdly said that malleability is "indeterminate fluidity." Remembering that the three states of matter are all of them continuous with reference to one another, that there is no sharp dividing lines between the solid, the liquid, and the gaseous states, it may be said that great pressure, or great force exerted in tension, simply brings the matter immediately under pressure into a changing state which is the bridge or link between two different states of matter.

Another property of matter of less importance to us here is elasticity, the power quite common to many metals, but possessed in widely different degree by different metals, of returning after elongation or compression to their original shape and form. A bar of steel may be drawn to greater than its original length, or it may be compressed to less than its original length, and yet when the force is removed it will return accurately to the first dimensions. If stretched or compressed beyond certain limits, it fails to return; it is said to have passed beyond the limit of elasticity. Here again we recognize a condition of freedom of motion within certain limits, and beyond that a condition of constraint; and applying it as before to the motion of the particles, we say that in the one case the particles have been moved out of their position but not so far as to prevent them returning to their original paths; the path may have been elongated, may have been contorted, but the original motion could reassert itself. In the second case, under strain the particles were compelled to take permanently new positions and to acquire to a certain extent new paths.

Color and lustre are surface qualities. Color depends merely upon the fact that the particles at the surface of a substance vibrate in unison with the medium from which they receive the light; light itself being a form of motion. But, inasmuch as white light is made up of many different rates of vibration, corresponding to the different colors which compose it, the vibrating particle receives or absorbs only the motion which corresponds to its own rate. The color represented in that motion will be lost from white light. The color corresponding to a different rate of motion, and therefore not absorbed, will be thrown back or reflected from the metallic surface, and the surface will appear to us to have a corresponding color. Copper is red because it absorbs from sunlight or white light all rays but the red and throws the red rays only back to the eye. Most metals absorb little of the motion of light, and throw most of it back. They are usually, therefore, of whitish or grayish color. Lustre is merely reflection of the light as a whole. A metal with an unpolished surface breaks up the light and reflects it in many different directions. In a polished surface all particles are lying virtually in the same plane, and all rays of light are thrown from that plane at the same angle. There is a difference to be noted, however, between the true lustre and simple reflection. Lustre is a quality of reflection, and is easier to name than to describe. Illustration will perhaps make my meaning clearer. We have only to think of the difference between the reflection of a plain glass mirror, on the one hand, and the lustre of polished lead or iron on the other. A slight element of color is introduced, perhaps, into the reflection, and this is the characteristic element in lustre.

The subject of alloys cannot be omitted from a discussion of the properties of metals. By an alloy we mean a mixture, in its simplest sense, of two metals; but the term mixture can by no means be used without qualification. It is, indeed, begging the question to use such a term, because the nature of alloys is one of the profoundest subjects of discussion in chemistry and physics. Are alloys mere mixtures, such as may be made of ink and water, or are they chemical compounds, such as occur when sulphur and iron are heated together? Some metals, when melted together and then cooled, show properties differing so greatly from those of either of the original metals that we can hardly believe them to be merely mixtures of the two. The alloy instead of melting at a temperature which is a mean of the melting-points of the two metals proportionate to their weight, generally melts at a lower point than this mean. Its powers of conducting heat and electricity, its relative volume, its solubility in acids, a large number of its properties, chemical and physical, are different from those of either of the original metals, and different from the mean quality of these metals with reference to the proportions taken in the alloy. Also it happens in many cases that, with certain definite proportions of two or more metals, we have alloys of new and remarkable properties; while a slight variation in the proportions, instead of giving merely proportionate change in quality, gives an alloy very little resembling the first. Now, this definiteness of properties corresponding to definiteness of composition is the characteristic and the test of chemical combination. The subject, however, is too wide a one to follow here. There are arguments, though fewer, in favor of the belief that the alloys are merely mixtures, but a compromise view is, perhaps, the most reasonable. We cannot escape the conclusion that chemical combination occurs in many cases, but the difficulties on the whole are simpified by assuming that alloys which are definite compounds may themselves dissolve in an excess of one or the other metal in the alloy, as many chemical compounds dissolve in the separate ingredients of the compound. And such an explanation would probably meet most cases where true chemical compounds are not formed.

There is one other point concerned here. There is a phenomenon known as allotropism, which very much modifies the qualities of elements and compounds. By allotropism we understand, referring again to our molecular hypothesis, an arrangement of the atoms within the molecule, such that the same substance, as iron or silver, may, without changing its chemical nature at all, exhibit very great difference in physical properties. Allotropism may even make changes in chemical properties, but the distinction is that the change is always a temporary one, and that the allotropic form of matter can always be brought back, and generally by simple means, to the original state of the same matter. For example, common phosphorus is a yellowish, wax-like solid, which melts at the temperature of the hand, and dangerous accidents have resulted from its handling. Red phosphorus is a substance obtained by merely heating common phosphorus with exclusion of oxygen. This second form of phosphorus is a red powder, requiring a temperature much above that of boiling water before it will burn in the air, being entirely safe to handle, not acted upon by chemicals in the same way, and practically harmless, while common phosphorus is a deadly poison. And yet we have merely to heat red phosphorus to a certain temperature in the air and it passes back at once to the state of common phosphorus. Oxygen, the familiar gas of the air, is readily converted into another gas, called ozone, which differs in a most marked degree from oxygen, and yet at a certain temperature it passes back into oxygen. We have in these cases, then, a simple change of physical quality without the slightest change of chemical integrity. The substance could be converted in a closed vessel from one state to the other and back again, nothing being added and nothing being taken from it.

Just as we have these cases of non-metallic allotropic bodies which I have given for the purpose of illustration, so we have among the metals some remarkable cases of allotropism. Tin under the action of great cold takes the condition of a grayish brittle substance, in which the original metal would not be recognized; yet there is no difficulty in bringing this back to the original tin. Silver, antimony, copper, and other metals also form allotropes.

Not to dwell further upon this subject, there is reason for believing that in alloys many metals may exist in the form of their allotropes, and so even if these alloys were mere mixtures, we should expect them to have qualities different from those of the metals concerned, or from the mean qualities of the metals. Whatever latitude we give to chemical or physical interpretation of the phenomena presented by alloys, it is certain that in the main changes of quality go with changes of proportion. Considering the number of the metals, the readiness with which the qualities of the alloys change with the proportions of these metals, and the infinity of permutations thus possible, the task of outlining the properties of all possible alloys is an infinite one. You will see, from the very brief treatment made of some of the properties of metals that the study of the alloys themselves offers a field for life-long investigation. However, what one knows practically of the metals may be applied at once in the study of their alloys, and I may hope, at least, that the few facts which I have passed in review before you, and the attempt that I have made to connect them and marshal them, so to spcak, under a comprehensive theory, may not be without some element of practical value to you.

